# Flexible 2D Structure Formation of [C_1_C_1_Im][Tf_2_N] on Ag(111)

**DOI:** 10.1002/cphc.202500163

**Published:** 2025-05-25

**Authors:** Afra Gezmis, Timo Talwar, Manuel Meusel, Andreas Bayer, Florian Maier, Hans‐Peter Steinrück

**Affiliations:** ^1^ Lehrstuhl für Physikalische Chemie II Universität Erlangen‐Nürnberg Egerlandstr. 3 91058 Erlangen Germany

**Keywords:** Ag(111), ionic liquids, scanning probe techniques, wetting layer, solid catalyst with ionic liquid layer

## Abstract

The first layer of the ionic liquid (IL) 1,3‐dimethylimidazolium bis[(trifluoromethyl)sulfonyl]imide ([C_1_C_1_Im][Tf_2_N]) on Ag(111) is studied under ultrahigh vacuum using scanning tunneling and atomic force microscopy, and the observed results are compared to the behavior found on Au(111), Cu(111), and Pt(111). On Ag(111), two stable phases are observed, a stripe phase and a hexagonal phase. The hexagonal phases on Ag(111), Au(111), and Pt(111) exhibit a similar, checkerboard‐like structure with surface areas per ion pair (IP) ranging from 0.77 to 0.85 nm^2^. In contrast, the striped phases behave differently: While for Au(111) and Cu(111), similar surface areas per IP as for the hexagonal phases were observed, the area per ion pair for Ag(111) varies from 0.50 to 1.14 nm^2^. This broad range results from a large variation of next neighbor intra‐ and interrow distances. These very substantial differences are attributed to the specific interplay of lateral interactions within IL rows and vertical interactions with the substrate. The pronounced polymorphism observed for [C_1_C_1_Im][Tf_2_N] on Ag(111) suggests that this system represents a special case, with the weakest adsorbate/substrate interaction among the studied systems, due to the absence of covalent interactions as compared to Cu(111) and only moderate van der Waals interactions as compared to Au(111).

## Graphical abstract

The first layer of the ionic liquid (IL) [C_1_C_1_Im][Tf_2_N] on Ag(111) displays a stripe phase and a hexagonal phase. In contrast to other (111) surfaces, the stripe phase on silver shows a large variation in intra‐ and interrow distances, which is attributed to the specific interplay of lateral interactions within IL rows and a comparably weak vertical interaction with the substrate.

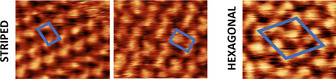

## 1 Introduction

The success story of ionic liquids (ILs) is evident from the growing number of publications over the last decades.^[^ ^1^, ^2^, ^3^ ^]^ The seemingly endless variety of combinations of anions and cations, and therefore, properties of the resulting ILs gave rise to studies in an enormous range of possible applications.^[^ ^4^, ^5^ ^]^ These are nearly as variable as the ILs themselves, with studies reported on their use in pharmaceutical industry,^[^ ^6^, ^7^, ^8^, ^9^, ^10^ ^]^ nuclear fuel processing,^[^ ^11^, ^12^, ^13^, ^14^, ^15^ ^]^ battery technology,^[^ ^16^, ^17^, ^18^, ^19^, ^20^ ^]^ lubrication,^[^ ^21^, ^22^, ^23^ ^]^ electrochemistry,^[^ ^24^, ^25^, ^26^, ^27^, ^28^, ^29^, ^30^, ^31^, ^32^, ^33^, ^34^ ^]^ separation,^[^ ^35^, ^36^, ^37^, ^38^, ^39^ ^]^ and in catalysis.^[^ ^40^, ^41^, ^42^, ^43^, ^44^, ^45^, ^46^, ^47^, ^48^, ^49^ ^]^ In the latter area, two novel promising concepts were introduced, that is, “supported IL phase” (SILP)^[^ ^43^, ^46^, ^48^ ^]^ catalysis and “solid catalyst with IL layer” (SCILL).^[^ ^49^ ^]^ In both concepts, a thin IL layer covers a porous support material. While in SILP, a catalyst complex is homogeneously dissolved in the IL film, in SCILL, the immobilized heterogeneous catalyst (typically metal nanoparticles on a support) is covered by a thin IL layer. To understand the working principles of SCILL, fundamental knowledge on the IL/metal interface is therefore of great importance.

The extremely low vapor pressure of ILs^[^ ^50^ ^]^ allows for the use of ultrahigh vacuum (UHV)^[^ ^51^, ^52^, ^53^ ^]^ and a variety of spectroscopic surface science techniques, including X‐ray photoelectron spectroscopy (XPS), reflection‐adsorption infrared spectroscopy, and UV photoelectron spectroscopy (see Refs. [3, 54, 55, 56] and references therein). Scanning tunneling (STM) and atomic force microscopy (AFM) complement the spectroscopic methods and provide additional, molecularly resolved real‐space insights on the IL layer in contact with its support.^[^ ^21^, ^28^, ^57^, ^58^, ^59^, ^60^, ^61^ ^]^ STM studies on a single frozen layer of 1‐octyl‐3‐methylimidazolium bis[(trifluoromethyl)sulfonyl)imide ([C_8_C_1_Im][Tf_2_N]) on Au(111)^[^ ^60^ ^]^ confirmed the checkerboard‐like structure of this IL, which was first suggested by Cremer et al.^[^ ^56^ ^]^ at room temperature (RT) using XPS. In this first layer, the so‐called wetting layer, the imidazolium rings of the cations and the [Tf_2_N]^−^ anions are adsorbed next to each other in direct contact to the metal, with the nonionic octyl chains of the cation and the CF_3_‐groups of the anion protruding away from the Au(111) surface. At RT, the ions become highly mobile, and the ordered 2D layer melts between 330 and 350 K, that is, about 40 K above the IL's bulk melting point of 295^62^–299^63^ K, as shown by AFM (see Supporting Information provided by Meusel et al.^[^ ^61^ ^]^). Combining STM and AFM, Meusel et al. investigated the wetting layer (WL) of the IL 1,3–dimethylimidazolium bis[(trifluoromethyl)sulfonyl)imide ([C_1_C_1_Im][Tf_2_N]) on Au(111) and found at low temperature (110 K) the coexistence of a striped and a hexagonal phase.^[^ ^61^ ^]^ Both phases have anions and cations lying next to each other in direct contact with the metal surface. Upon increasing the temperature, an overall hexagonal layer was formed,^[^ ^62^, ^63^ ^]^ which could be detected in STM and AFM even up to RT.^[^ ^61^ ^]^

Upon annealing a WL of the same IL on Cu(111) from 110 to 300 K, Adhikari et al.^[^ ^58^ ^]^ saw the transformation from a striped to a hexagonal and further to a pore‐type honeycomb phase in STM and AFM. This transformation was accompanied by partial IL decomposition starting at 275 K, as observed by XPS. Upon further annealing to 350 K, the IL film fully decomposed, leaving only small disordered islands on the Cu(111) surface behind. The striped phase at 200 K was reported to be a checkerboard‐like structure with cation and anion in direct contact with the surface. In contrast, for the hexagonal and the honeycomb phases, a different arrangement of the ions in a sandwich‐type adsorption motif was suggested, namely, ion pairs (IPs) of cation and anion stacked on top of each other with next neighbor sandwiches having alternating vertical orientations.^[^ ^58^ ^]^

On the even more reactive Pt(111) surface, [C_1_C_1_Im][Tf_2_N] was reported to form a disordered layer upon deposition at temperatures below 170 K. Annealing to 200 K induced the formation of ordered, stripe‐like or hexagonal structures.^[^ ^64^ ^]^ Above 250 K, decomposition of the IL was deduced from spectral changes in XPS^[^ ^64^ ^]^ and the appearance of bright protrusions next to ordered hexagonal regions in STM. At 300 K, 50%–60% of the IL layer was found to be decomposed; thereby, the anion‐related reaction products desorbed instantaneously and the cation‐related products remained on the surface. This led to a partial passivation of the surface, enabling the remaining IL to still be adsorbed intact until ≈350 K. The decomposition process continued upon further annealing until 375 K, when the CF_3_ anion signals in XPS abruptly dropped to zero indicating full IL decomposition.^[^ ^64^ ^]^

Similar microscopy studies for IL layers on silver are still missing despite the fact that this substrate plays an important role: Palladium–Silver (Pd–Ag) catalysts are known to enhance the selectivity in industrially relevant hydrogenation reactions such as the selective hydrogenation of acetylene to ethylene. It has been proposed that Ag lowers the amount of adsorbed hydrogen, and thus, suppresses the second hydrogenation step to ethane.^[^ ^65^ ^]^ Moreover, density functional theory (DFT) calculations show that the adsorption energy of ethylene on Pd–Ag surfaces decreases with increasing amount of Ag, while the reaction barrier for the hydrogenation from ethylene to ethane increases and thus this process is not favored.^[^ ^66^ ^]^ For the selective hydrogenation of 1–octyne to 1‐octene, a 10%Pd 90%Ag alloy on Al_2_O_3_ with one monolayer of 1‐butyl‐3‐methylimidazolium dicyanamide ([BMIM][DCA]), corresponding to 14 wt%, has shown to successfully suppress the second hydrogenation step from 1‐octene to octane (84% 1‐octene yield at 91% 1‐octyne conversion).^[^ ^67^ ^]^ Notably, the role of Ag in this SCILL system is not understood yet and warrants detailed investigations. However, prior to addressing the complex Pd–Ag alloy system, the interaction with the pure metals has to be understood.

**Figure 1:**
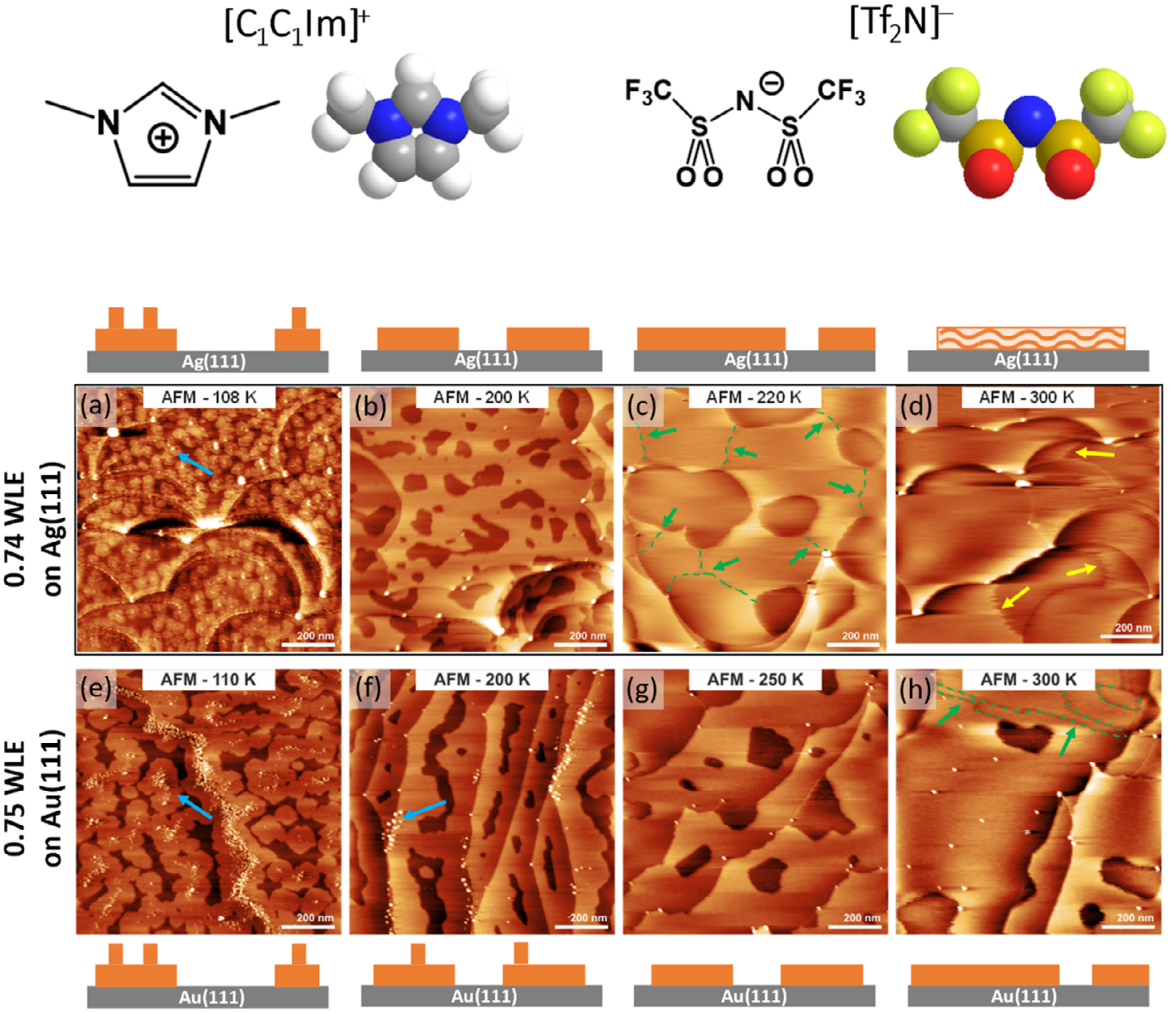
AFM images (1000 × 1000 nm) of [C_1_C_1_Im][Tf_2_N] films of 0.74 WLE on Ag(111) (top, a–d) and 0.75 WLE on Au(111) (bottom, e–h) deposited at LTs (<170 K), and measured at 108 K/110 K, 200 K, 220 K/250 K, and 300 K. Second layer species found at 108 K on Ag(111) and Au(111), and at 200 K on Au(111) are marked by blue arrows. Visible contact lines of two or multiple domains (green arrows and dashed lines, see also Figure S1, Supporting Information) show that on Au(111) the film is stable at 300 K, while the contact lines on Ag(111) were only observed at 220 K and not at 300 K, indicating that the IL film starts melting between 220 and 300 K. The stripy appearance of island edges (yellow arrows) on Ag(111) at 300 K indicate melting of the Il film. The small bright protrusions, which are mainly observed on Au(111), are attributed to defects. Schematic representations of the IL films on the Ag(111) and Au(111) surfaces are given above and below the AFM images, respectively. The IL structure is illustrated at the very top of the figure.

In order to gain further insights of the Ag/IL interactions, we therefore investigated the first layer of the model IL [C_1_C_1_Im][Tf_2_N] adsorbed on a clean Ag(111) surface by STM and AFM under UHV conditions. The IL structure is shown at the very top of **Figure** **1**. Comparing the structure formation of this IL on Ag(111) to that on other hexagonally close‐packed surfaces like Au(111),^[^ ^61^, ^68^ ^]^ Cu(111),^[^ ^58^ ^]^ and also Pt(111) showed how differences in IL/metal interactions, or the lack thereof, can modify the flexibility of the formed IL structures.

## 2 Results and Discussion

We investigated ordered 2D films of [C_1_C_1_Im][Tf_2_N] in the subWL regime; thereby, a coverage of 1 WL corresponds to 100% of the surface covered with IL ions next to each other in direct contact with the surface in a checkerboard‐like structure. The films were prepared by evaporating different doses of IL on a freshly cleaned Ag(111) surface following a procedure described previously.^[^ ^61^ ^]^ Physical vapor deposition was performed onto the sample at RT or at LT (LT, that is, *T*_s_ < 170 K), followed by immediate transfer to the STM/AFM, where it cooled down to ≤110 K within 8–15 min for optimum imaging conditions. The experimental conditions and settings of the STM and AFM experiments are provided in Table S1, Supporting Information.

In the following, we discuss AFM images of a subWL film on Ag(111) in comparison to a similar film on Au(111). AFM was chosen for these studies as the interaction of the tip with the IL film, in particular for second layer species or at higher temperatures, is weaker than in case of STM, which allows for better imaging.^[^ ^61^ ^]^ We first deposited 0.74 WL equivalents (WLE) of IL onto a clean Ag(111) surface at LT. Note that our evaporator was calibrated on Au(111), where a dose of 1.0 WLE yields a closed WL, covering the whole surface. In the subWL regime, this can directly be translated to the surface coverage, if no changes in IL density on the surface occur, for example, due to structural changes.

Figure 1 (top) shows the corresponding AFM images of a [C_1_C_1_Im][Tf_2_N] film on Ag(111) with a nominal coverage of 0.74 WL, measured at different temperatures. In Figure 1 (bottom), images of the corresponding film of the same IL on Au(111) are depicted (note that these data have not been published yet). After deposition, the AFM measurements on both surfaces at ≈110 K show the formation of small 2D islands with bright protrusions on top of their center, which are assigned to IL in the second layer (blue arrows). On Ag(111), upon annealing to 200 K, we observe the ripening of the IL islands, yielding a flat 2D layer. On Au(111), ripening is also observed, but the second layer IL species did not disappear completely. Upon further annealing to higher temperatures (220 K for Ag(111), 250 K for Au(111)), large interconnected 2D IL areas are observed on both surfaces, with some regions of uncovered surface (darker areas in the images) in between. This behavior has already been reported for this IL on Au(111).^[^ ^68^ ^]^ The small bright protrusions, which are mainly observed on Au(111), are attributed to defects. On Ag(111), the 2D IL film melts upon heating to 300 K, as is deduced from two observations: (1) it is still possible to resolve the contact line of two or multiple domains at 220 K (see green arrows and dashed lines in Figure 1c; for more details, see also Figure S1, Supporting Information), while it is not at 300 K (see Figure 1d). (2) The IL island edges exhibit a washed‐out appearance after heating to 300 K (see yellow arrows in Figure 1d). This melting process can be followed in more detail in Figure S2, Supporting Information, from which a melting point around 280 K is deduced. On Au(111), melting occurs only above 300 K, as concluded from the observation of sharp contact lines and island edges at 300 K in Figure 1h, which has a similar appearance as in Figure 1c for Ag(111) at 220 K. The lower melting temperature on Ag(111) is further confirmed by the observation that it was not possible to obtain molecularly resolved AFM images on Ag(111) at 300 K, in contrast to reports on Au(111), where the molecular structures of this IL were resolved even at 300 K.^[^ ^61^ ^]^ A schematic representation of the IL films on the Ag(111) and Au(111) surfaces is shown above and below the AFM images in Figure 1, respectively. The lower melting point of the IL WL on Ag(111) could be an indication for a weaker interaction of the IL with Ag(111) as compared to Au(111). The same conclusion is derived from the by ≈10 K lower desorption temperature on Ag(111) than on Au(111), namely ≈425 versus ≈435 K, respectively (see Figure S3, Supporting Information).

**Figure 2:**
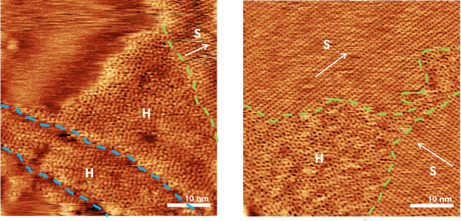
AFM images (50 × 50 nm) of 0.77 WLE (left) and 0.50 WLE (right) of [C_1_C_1_Im][Tf_2_N] on Ag(111), deposited at LT (<170 K), after annealing to 200 K for 10 min. The films display coexisting hexagonal phases (H) and striped phases (S); phase boundaries are marked by green dashed lines and step edges by blue dashed lines. Stripe directions are marked with white arrows.

In the next step, we analyze the subWL films of [C_1_C_1_Im][Tf_2_N] on Ag(111) in more detail. **Figure** **2** shows two AFM images after deposition of 0.77 and 0.50 WLE IL below 170 K, followed by annealing to 200 K. We observe two different phases, namely a striped phase (S) and a hexagonal phase (H). Notably, the striped phase is the majority phase and the hexagonal phase is the minority phase. The AFM images in Figure 2 have been selected on purpose such that both phases can be compared. The phase boundaries between H and S are marked with green dashed lines. The H phases tend to form near step edges of the underlying metal surface (left image; blue dashed lines). As denoted, the H phase on Ag(111) was observed only in rare cases and only in very small quantity and size. Annealing the sample for a longer time or increasing the annealing temperature did not change the likelihood of observing an H phase. No H domains were observed, when the IL was evaporated onto the surface at RT, and no coverage dependency of the H phase was found (see Table S3, Supporting Information). Still, the possibility of small H domains on these samples cannot be excluded as the complete sample surface cannot be probed by scanning probe microscopy (SPM).

**Figure 3:**
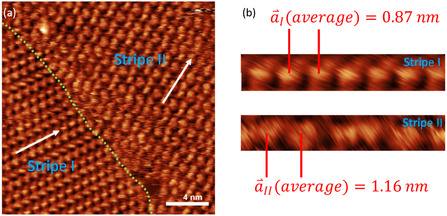
a) STM image (20 × 20 nm) of 0.50 WLE of [C_1_C_1_Im][Tf_2_N] on Ag(111), deposited at RT and measured at 106 K. The STM image shows the two sub‐phases Stripe I (S‐I) and Stripe II (S‐II) of the S phase. The contact line of the domains is marked by the green dashed line. The average intrarow distance |$\vec{a}$| for S‐I is 0.87 nm, and for S‐II is 1.16 nm. The stripe direction $\vec{a}$ is marked by white arrows. b) Exemplary, enlarged cutouts of single stripes from domains S‐I and S‐II. S‐I shows a highly regular repeating pattern of protrusions, while S‐II shows more nonregularities.

To analyze the different phases in the subWL range in more detail, we switched to STM, which for the here‐studied systems and conditions yielded better image quality with higher resolution. **Figure** **3**a shows an STM image with two domains of the S phase. The domain boundary is marked with a green dashed line. The directions of the stripes are marked with white arrows; notably, they do not show any systematic relation to the substrate orientation. Measuring the average distances of the protrusions along the stripe direction ($\vec{a}$ vector) in Figure 3a yields 0.87 nm for phase S‐I (bottom left) and 1.16 nm for phase S‐II (top right). Cutouts from single stripes of both domains are shown in Figure 3b. Phase S‐I is better resolved and shows a regular repeating pattern of visible protrusions. The appearance of the protrusions in phase S‐II is not as regular, which might be due to orientational differences of the ions in this phase, possibly due to mismatch with the underlying Ag(111) substrate. Due to the larger size of the anion, we tentatively assign the protrusions to the anions, similar to the observation of this IL on the other reported surfaces.^[^ ^58^, ^61^ ^]^ The analysis of a large number of images shows that the rows are nonsystematically shifted with respect to each other. For [C_1_C_1_Im][Tf_2_N] on Au(111), Meusel et al. have already reported a variation of the row‐to‐row alignment for a striped structure, where the rows were found to be shifted statistically along the row direction, forming line defects in the stripe order.^[^ ^61^ ^]^

**Figure 4:**
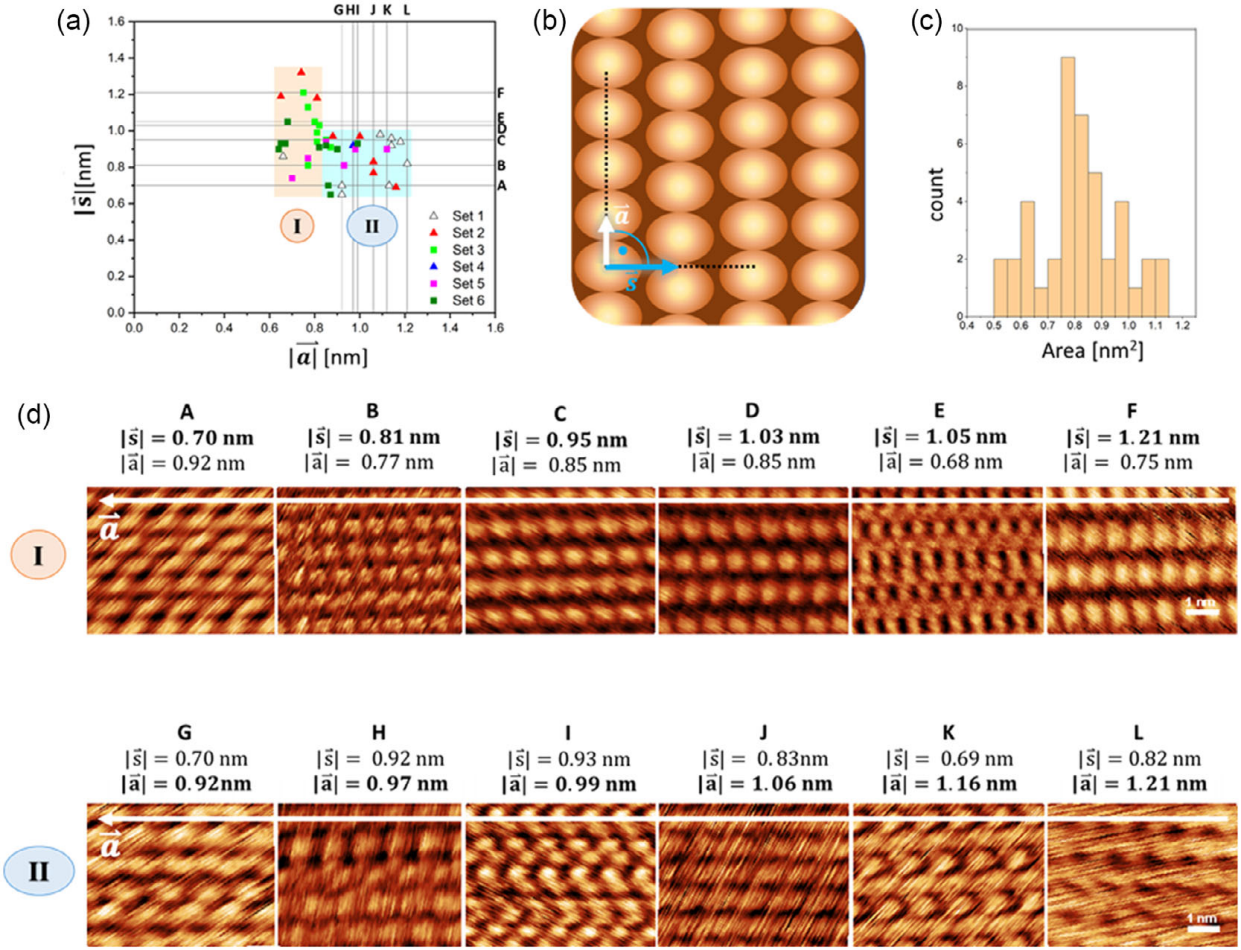
a) Relation of the next neighbor (intrarow) distances |$\vec{a}|$ measured along the stripes of the S structure and the stripe‐to‐stripe (interrow) distance $\left|\vec{s}\right|$ perpendicular to $\vec{a}$. The different datasets vary in evaporation temperature (RT: empty symbols, LT (<170 K): full symbols), coverage or measurement type (STM: square, AFM: triangle). Explanation of the conditions of each set is given in the Table S2, Supporting Information; note that no systematic variation with any of these parameters is found. b) Schematic explanation of the measured vectors/distances $\left|\vec{a}\right|$ and |$\vec{s}|$; c) Distribution of the areas formed by the vectors $\vec{a}$ and $\vec{s}$ measured in (a). d) Representative STM or AFM images of different observed structures: Values A‐F (top series) and G‐L (bottom series) above the images indicate the corresponding |$\vec{s}|$ and $|\vec{a}$| values in (a). The images are ordered by increasing $\left|\vec{a}\right|$ (bottom, G‐L) and |$\vec{s}|$ (top, A‐F) values from left to right and attributed to domain type S‐I (top) and domain type S‐II (bottom), respectively. In each image, the direction of |$\vec{a}|$ is marked by a white arrow. We assume that the visible protrusions represent the anion while the cation is not visible due to its flat‐lying adsorption geometry.

For the detailed characterization of the S phases on Ag(111), we measured two characteristic distances (vectors) for a large number of images: The next neighbor distance between periodically repeated protrusions along the stripe direction is described by $\left|\vec{a}\right|$, and the distance of adjacent rows by $\left|\vec{s}\right|$, which is perpendicular to $\left|\vec{a}\right|$ (both distances are determined as average values for each image). The values of $\left|\vec{a}\right|$ and |$\vec{s}|$, and the relative position of neighboring rows, that is, the shift along the row direction, show a large nonsystematic variation, which cannot be correlated to specific differences in preparation or measurement conditions (data sets 1–6 in Table S2, Supporting Information). In an attempt to characterize the observed structures, we plotted |$\vec{s}|$ versus $\left|\vec{a}\right|$ for a large number of analyzed images in **Figure** **4**a. Notably, these data sets vary in deposition temperature (open symbols for IL evaporation on a RT surface and full symbols for evaporation on a LT surface), IL coverage or measurement type (AFM: triangles, STM: squares), but no systematic correlation could be found. In a next step, we calculated the areas formed by vectors $\vec{a}$ and $\vec{s}$ in Figure 4a. The results again show a large variation, with areas ranging from 0.50 to 1.14 nm^2^ (see Figure 4c). We, therefore, continue with the analysis of the relation between the interrow distance $\left|\vec{s}\right|$ and the intrarow distance |$\vec{a}|$ as shown in Figure 4a. By doing so, we can roughly categorize the observed structures into two groups, I and II, which we tentatively correlate with the phases S‐I and S‐II observed in Figure 3 (notably, the differentiation of these two phases is somewhat arbitrary but allows for a clearer description of our observations).

For phase S‐I, which is characterized by |$\vec{a}$| values from ≈0.65 to ≈0.90 nm, we observe a large variation in interrow distance $\left|\vec{s}\right|$, from 0.65 to 1.32 nm (Figure 4a, shaded in orange). Figure 4d shows exemplary cutouts from the STM and AFM images evaluated in Figure 4a,c. These cutouts are aligned horizontally along the direction of the $\vec{a}$ vector of the first stripe (white arrow); reference to Figure 4a (A to F) and the corresponding values of |$\vec{a}$| and $\left|\vec{s}\right|$ are given on top of each image. Comparing the different images illustrates the scatter of the stripe distances $\left|\vec{s}\right|$. The visual appearance and the |$\vec{a}$| values of these stripes of typically below ≈0.90 nm correspond to the one observed for the domain S‐I in Figure 3a. Occasionally, we also observe double stripes (see in Figure 4d, e.g., E), where the interrow distances periodically alternate between smaller and larger |$\vec{s}$| values (in these cases, the average value is denoted in Figure 4).

Phase S‐II is characterized by |$\vec{a}$| values from ≈0.90 to ≈1.20 nm (Figure 4a, shaded in blue, and Figure 4d, bottom), and a much smaller variation of the interrow distance |$\vec{s}$|, that is, 0.65 to 0.98 nm (note that double or even triple rows are observed here as well, e.g., in G and K). The appearance and the |$\vec{a}$| values are in the range of domain S‐II in Figure 3a. Figure 4d (bottom) shows cutouts from exemplary images with reference to Figure 4a given on top (G to L).

To summarize, we group the observed striped domains in Figures 3 and 4 in two types of phases, S‐I and S‐II. Phase S‐I is a striped structure formed by single rows (and in some case double rows) of IPs with a comparably small intrarow distance $\left|\vec{a}\right|$ of 0.78 ± 0.13 nm, but a variable interrow distance of 0.95 ± 0.30 nm. In contrast, phase S‐II with a larger intrarow distance $\left|\vec{a}\right|$ of 1.05 ± 0.15 nm shows a much smaller variation in the interrow distance with |$\vec{s}$| values of 0.82 ± 0.17 nm. The smaller variation could be due to the less regular arrangement of ions within the row, as shown in Figure 3b. Notably, despite the fact that value ranges of $|\vec{s}$| and |$\vec{a}$| are similar, these are not simply exchangeable with each other, as the interrow values |$\vec{s}$| are not determined from repeating protrusions as is done for the intrarow values |$\vec{a}$|, but by the distance of rows, which are shifted to each other very differently in the different structures. The variation in appearance of the stripes suggests conformational changes from one row to the other, which result in the variety of intrarow next neighbor distances and the observed polymorphism.

For a pyrrolidinium‐based IL, [C_4_C_1_Pyr][Tf_2_N] (1‐butyl‐1‐methylpyrrolidinium bis[(trifluoromethyl)sulfonyl]imide; notably, this IL is also denoted as [BMP]^+^[TFSA]^−^ or [C_4_C_1_Pyr][NTf_2_]) on highly ordered pyrolytic graphite (HOPG), Buchner et al.^[^ ^59^ ^]^ also reported a striped structure and proposed that the interaction strength between ions within a row can be notably different compared to that between adjacent rows, allowing for a variation of the row distances. It should be noted that in the system studied by Buchner et al. only one striped phase was found and the variation in $\left|\vec{s}\right|$ values was smaller. For [C_2_C_1_Im][Tf_2_N] on Ag(111), an IL with a slightly larger cation then the one used here, Uhl et al. also reported variations in unit cell dimensions on different locations on the surface,^[^ ^60^ ^]^ with the magnitude of the variations in the range of the standard deviation. Interestingly, no such deviations were reported for their investigations of [C_8_C_1_Im][Tf_2_N] on the same surface, indicating that lateral van der Waals interactions of the alkyl chains in the WL contribute to a stabilization of the structure on the Ag(111) surface.

Another interesting and even more relevant study was performed by Eschenbacher et al. for [C_4_C_1_Pyr][Tf_2_N] on Pd(111) by infrared reflection absorption spectroscopy and molecular dynamics simulations.^[^ ^69^ ^]^ From the latter, the authors deduce the completion of a full WL for 90 IPs per 99 nm^2^, which can be densified up to 144 IPs per 99 nm^2^, that is, by 60%. This densification occurs via reorientation and changes in the conformation of cations and anions leading to a decrease of the required surface area per IP, from 1.10 to 0.69 nm^2^/IP. This behavior indicates that the adsorption energy of the ions on Pd(111) does not strongly depend on the adsorption geometry of the ions, and thus, larger lateral interactions induce the orientational and conformational changes toward less space‐demanding motifs. One should note here that the pyrrolidinium ring is not aromatic and thus does not strongly interact with the reactive Pd(111) surface.

Considering these reports, we propose the following interpretation for the very large variety of different structures observed for [C_1_C_1_Im][Tf_2_N] on Ag(111). We assign them to ordered IL phases with different adsorption motifs in terms of orientation and conformation of the ions. The motifs have quite similar adsorption energies, which leads to the coexistence of the different phases, that is, a pronounced polymorphism. The large variation of surface area per IP for the different motifs indicates that the interaction to the substrate has a low site specifity, and also lateral interactions do not seem to play a major role. This behavior is partly attributed to the short methyl chains of the [C_1_C_1_Im]^+^ cations, which—in contrast to systems with longer alkyl chains (see above)—provide only small possibilities for stabilizing specific motifs by intra‐ and interrow van der Waals interactions, yielding altogether very similar adsorption energies and thus a large variation of the interion distances.

**Figure 5:**
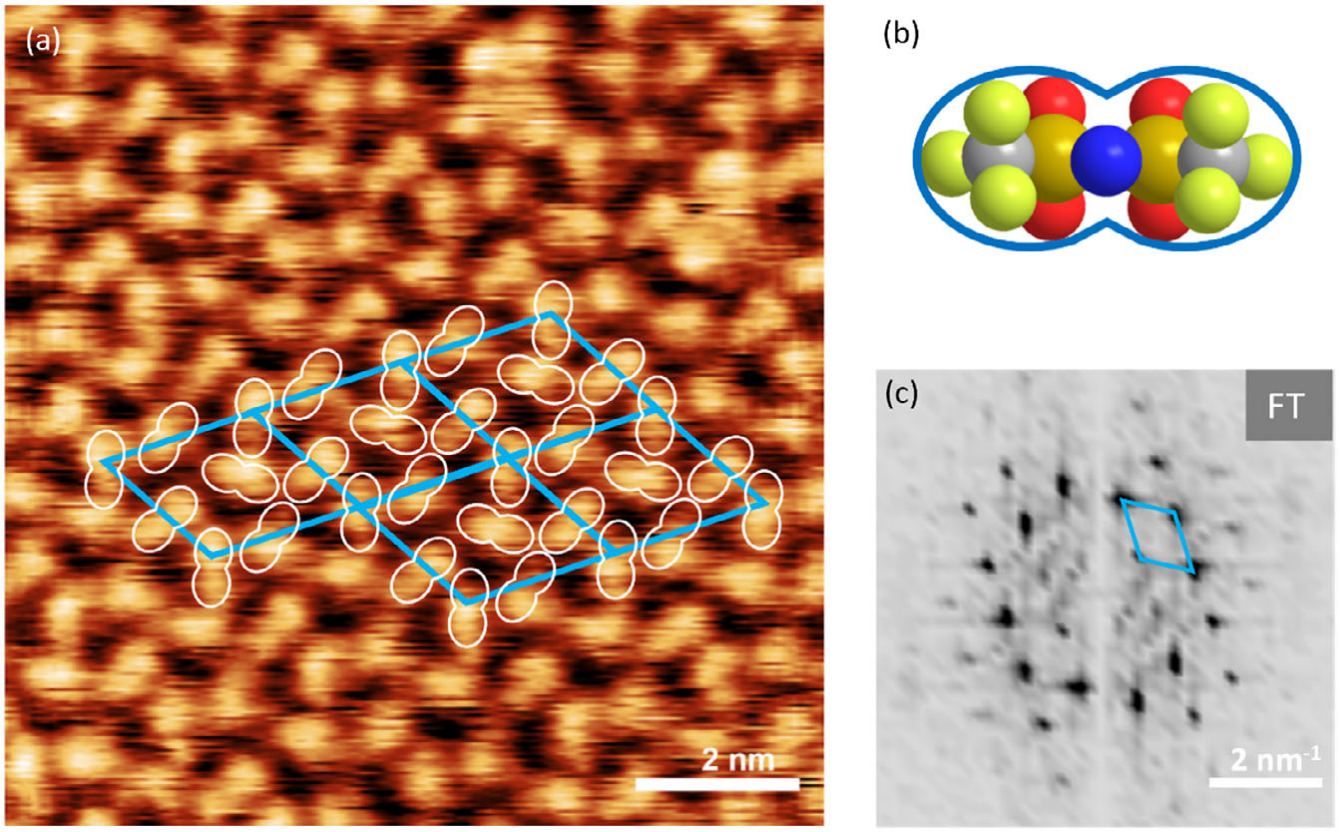
a) STM image of the hexagonal phase of a [C_1_C_1_Im][Tf_2_N] film (0.94 WLE), deposited onto Ag(111) at <170 K, after annealing to 300 K for 10 min. The nonprimitive unit cell is marked in blue. The visible elongated protrusions marked by white contours (called “peanuts”) are assigned to the anion positions. The cation positions cannot be assigned, as they are not visible due to their flat‐lying adsorption geometry. b) explains the correlation of the [Tf_2_N]^−^ anion to the “peanut” shape. c) shows the FT of the STM image of (a) verifying the hexagonal structure. The reciprocal unit cell is also marked in blue.

We next turn to the minority phase H, which in contrast to the stripe phase has a very uniform appearance. **Figure** **5** (left) displays a molecularly resolved STM image of the H phase, which shows pronounced long‐range order. We can identify a nonprimitive unit cell (blue), which contains four bright protrusions. It is defined by vectors $\vec{a}$_H_ and $\vec{b}$_H_, with lengths of 1.92 ± 0.10 nm and 1.85 ± 0.04 nm, respectively, with an enclosed angle *γ* of 60°. These values are supported by the Fourier‐transformation (FT) of the image (Figure 5c). The resulting unit cell area is 3.08 nm^2^. We propose a model with four IPs per unit cell (blue) in a checkerboard‐like arrangement. We thereby assume that only the [Tf_2_N]^−^ anion is visible and that the cation cannot be imaged due to its flat‐lying adsorption geometry, similar to the situation for this IL on Au(111).^[^ ^70^ ^]^ The exact position of the cation can therefore not be determined from SPM images. The IP density is 1.30 IPs nm^−^^2^ and the surface area occupied by one IP is 0.77 nm^2^. The white elongated features in Figure 5 (which we refer to “peanuts”, see Figure 5b) mark the positions of the anions.

In order to obtain a more comprehensive understanding of the observed WL behavior of [C_1_C_1_Im][Tf_2_N] on Ag(111), we compare our results to similar investigations for other hexagonally closed‐packed surfaces, that is, Au(111), Cu(111), and Pt(111). While WLs of [C_1_C_1_Im][Tf_2_N] on Au(111)^[^ ^61^ ^]^ and Cu(111)^[^ ^58^ ^]^ have been studied in detail before by SPM, information on the structure and density of the WL on Pt(111) is very limited.^[^ ^64^ ^]^ We therefore address this surface in the following in a little more detail. The challenge on Pt(111) is that the temperature range, in which the formation of ordered layers occurs, partly overlaps with the onset of IL decomposition.^[^ ^64^ ^]^ Figure S4, Supporting Information (top), shows a series of STM images, which have been measured after deposition onto Pt(111) below 170 K, followed by annealing to 200 or 250 K for 10 min (for details on sample preparation and IL deposition see Massicot et al.^[^ ^64^ ^]^). The STM images predominantly show small to medium sized domains with hexagonal order (Figure S4, Supporting Information, bottom). The lattice vectors of the hexagonal domains have a length of vectors |${\vec{a}}_{H}|$ = 0.98 ± 0.07 nm and |$\vec{b}$_H_| = 0.96 ± 0.18 nm, with an enclosed angle of *γ* = 60 ± 3°, which results in a surface area per IP of 0.81 ± 0.19 nm^2^. In few instances, domains with a striped appearance (not shown) were observed, which were difficult to reproduce. We thus refrained from a further analysis.

**Figure 6:**
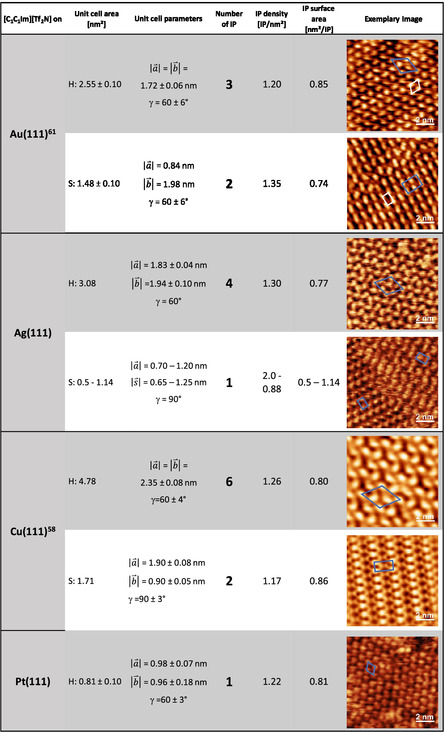
Comparison of the striped (S) and hexagonal (H) phases of [C_1_C_1_Im][Tf_2_N] on Au(111), Ag(111), Cu(111), and Pt(111). The different phases are compared concerning their unit cell area, unit cell parameters, number of IPs within the unit cell, IP density, and the surface area required to adsorb one IP. Exemplary STM or AFM images of each structure are shown on the right. The images for the H phase on Au(111) and for the H and S phases on Cu(111) were adapted from work done by Meusel and Adhikari,^[^ ^58^, ^61^ ^]^ respectively, under CC BY 4.0 license. The data for the S phase on Au(111) and for the H phase on Pt(111) are in line with data previously published by Meusel and Massicot,^[^ ^61^, ^64^ ^]^ respectively, but are shown here for the first time. Please note that the values for the IL/Au(111) system are adjusted to take into account the orientation of the visible protrusions (blue unit cell) and not just the primitive unit cell (white). Additionally, it is important to mention that on Cu(111) we observed a third structure (honeycomb structure) with the same unit cell parameters as the hexagonal structure.^[^ ^58^ ^]^ This structure has not been included as there is no comparable structure formed on the other surfaces.

In the following, we compare the density of the observed [C_1_C_1_Im][Tf_2_N] phases on Ag(111) to those reported previously on Au(111) and Cu(111), and also to those on Pt(111) reported in the previous paragraph. On all surfaces, striped and hexagonal phases have been observed, as is evident from **Figure** **6**. We start with comparing the hexagonal phases and find that the unit cell area (and the number of IPs) increases from 0.81 nm^2^ (1 IP) on Pt(111), to 2.55 nm^2^ (3 IPs) on Au(111),^61^ to 3.08 nm^2^ (4 IPs) on Ag(111), and 4.78 nm^2^ (6 IPs) on Cu(111);^[^ ^58^ ^]^ see Figure 6. Notably, the IP density, and therefore, the surface area per IP are very similar for all four systems, with values of 0.81, 0.85, 0.77, and 0.80 nm^2^/IP, respectively, that is, an average value of 0.81 ± 0.04 nm^2^/IP. For Au(111), Ag(111), and Pt(111), this similarity reflects the similarity of the checkerboard‐like structures of the wetting layer, with cations and anions in direct contact with the substrate. For Cu(111), this similarity is, however, quite surprising, since the IP have been proposed to not adsorb in a checkerboard‐like structure, but as alternatingly oriented sandwiches of cations and anions on top of each other.^[^ ^58^ ^]^ It should be noted that on Cu(111), upon annealing at 300 K for an extended period (≈16 h), [C_1_C_1_Im][Tf_2_N] forms a third structure, that is, the porous honeycomb structure. The honeycomb structure has the same unit cell parameters and unit cell area as the hexagonal structure, but differs in the number of IPs (5 vs 6, respectively), and therefore, also in IP density and the area required to adsorb one IP.^[^ ^58^ ^]^ This structure has not been included in this discussion, as there is no comparable structure formed on the other investigated surfaces.

The comparison of the S phases on the different surfaces shows some interesting similarities and differences. For Au(111) and Cu(111), the surface areas per IP are similar with values of 0.74 and 0.86 nm^2^/IP, respectively, that is, an average value of 0.80 ± 6 nm^2^/IP, respectively. Interestingly, this density is very close to that observed for the H phase (0.81 ± 0.04 nm^2^/IP; see above). The only exception is the stripe phase on the Ag(111) surface, where the surface area per IP varies strongly, from 0.5 to 1.14 nm^2^/IP.

This very different behavior for the S phase on Ag(111) and Au(111) is most surprising, considering the fact that both surfaces are chemically quite inert. There are, however, also some important differences. When comparing our results for these two surfaces, we observe a higher melting temperature and a more uniform striped phase on Au(111). This behavior is attributed to the complex interplay of IL/substrate interactions with the IL/IL interactions in the wetting layer. As the Au(111) surface is considered to be less reactive, reactivity cannot explain the observations. One major difference is that the Au(111) surface is more polarizable, due to the higher number of electrons per atom, and therefore, might bind charged molecules, such as IL ions, more strongly.^[^ ^71^ ^]^ Geada et al. reported that the large polarizability of Au(111) increases the bonding strength of charged ions absorbed on the surface.^[^ ^72^ ^]^ The increased interaction strength of [C_1_C_1_Im][Tf_2_N] on Au(111) as compared to Ag(111), might lead to a stronger corrugation of the surface potential, and thus, a higher site specificity on the Au(111) surface, which in turn could lead to a well‐defined interrow distance and unit cell of the adsorbed IL. In contrast, on Cu(111) and even more on Pt(111), covalent interactions due to the more reactive nature of the substrates potentially lead to a stronger stabilization of the WL on these surfaces.^[^ ^73^, ^74^ ^]^ In other words, the IL may be chemisorbed at specific adsorption sites on Cu(111) and Pt(111), which leads to the more well‐defined unit cells in the observed structures.

To conclude, we propose that [C_1_C_1_Im][Tf_2_N] on Ag(111) has the weakest interaction strength and also the lowest site specificity of the here‐studied surfaces, with very similar adsorption energies for the different adsorption motifs, which leads to more lateral freedom of the resulting structures. This behavior, together with only weak lateral van der Waals interactions between the IL moieties, as compared to ILs with longer alkyl chains, makes the reported striped phase of [C_1_C_1_Im][Tf_2_N]/Ag(111) a very unique case among the studied IL/metal systems, displaying a pronounced polymorphism, that is, coexistence of different structures with similar energies. Interestingly, the hexagonal phase on Ag(111) behaves similarly as on the Au(111) and Pt(111) surfaces, which could indicate a stabilization with a specific orientation of the ions in this structure. In order to understand the specific driving forces for these observations, one requires detailed DFT calculations, which are, however, out of the scope of the present study. Such calculations along with additional experiments will help to elucidate whether the observed flexibility of [C_1_C_1_Im][Tf_2_N] on Ag(111) is a general phenomenon, and whether it contributes to the reported outstanding performance of SCILL Pd–Ag catalysts in the selective hydrogenation of acetylene to ethylene.

## 3 Summary and Conclusion

We investigated the structure of the first layer of the IL [C_1_C_1_Im][Tf_2_N] on Ag(111) under UHV conditions. Using STM and AFM, we observed two stable phases, a striped phase, which is the majority phase, and a hexagonal phase, which is the minority phase. Both are assigned to checkerboard‐like structures with the cation and anion in direct contact to the metal surface. When comparing these structures to those reported for the same IL on Au(111), Cu(111), and Pt(111), we find interesting similarities and differences. The hexagonal checkerboard‐like phases on Ag(111), Au(111), and Pt(111) have very similar surface areas per IP, ranging from 0.77 to 0.85 nm^2^. In contrast, for the striped phases on Ag(111), Au(111), and Cu(111) significant differences are observed. While on Au(111) and Cu(111) with surface areas per IP ranging from 0.74 to 0.86 nm^2^, a similar packing density is observed as for the hexagonal phases, the packing density of the stripe phase on Ag(111) varies strongly, with surface areas per IP ranging from 0.50 to 1.14 nm^2^. This behavior results from a large variation of next neighbor intra‐ and interrow distances. The observed striped domains on Ag(111) are tentatively grouped into two types of phases, S‐I and S‐II. Phase S‐I is a striped structure formed by single rows (and in some case double rows) of IPs with a comparably small intrarow distance $\left|\vec{a}\right|$ variation of 0.78 ± 0.13 nm, but variable interrow distance |$\vec{s}$| of 0.95 ± 0.30 nm. Phase S‐II has a larger intrarow distance $\left|\vec{a}\right|$ variation of 1.05 ± 0.15 nm (also sometimes double or triple rows) and a much smaller interrow distance |$\vec{s}$| variation of 0.82 ± 0.17 nm. The variations in appearance of the stripes suggest conformational and orientational changes from one row to the other, which result in the variety in intrarow next neighbor and interrow distances and the observed polymorphism.

We propose that the very different behavior for the S phases on Ag(111) as compared to Au(111) and Cu(111) is due to the weaker adsorption strength of [C_1_C_1_Im][Tf_2_N] on Ag(111). While both Ag(111) and Au(111) are chemically quite inert, one expects stronger physisorption on Au(111) due to its higher polarizability, which is in line with the higher melting temperature and the higher desorption temperature of the IL for Au(111). On the other hand, Cu(111) and Pt(111) are known for being more reactive allowing for the formation of covalent bonds with the IL, which is reflected by partial decomposition of the IL upon heating.

## 4 Experimental Section

All experiments were carried out by constant‐current STM and noncontact AFM, using a Scienta Omicron VT‐AFM‐Q+‐XA instrument. The base pressure of this two‐chamber UHV system was <1 × 10^−10^ mbar. The STM images were recorded with a W tip using bias voltages from −1.5 to +1.2 V. The AFM images were recorded in noncontact mode. Frequency shifts ranging from −160 to −600 Hz, respective to the cantilever resonance frequency, typically around 300 kHz, were applied. The IL [C_1_C_1_Im][Tf_2_N] was synthesized as described in previous publications^[^ ^75^ ^]^ and deposited using a home build effusion cell at cell temperatures from 406 to 414 K.^[^ ^76^ ^]^ Prior to deposition, the IL was degassed for several hours. The IL coverage was controlled by checking the IL flux using a quartz crystal microbalance, which was calibrated before as described by Meusel et al.^[^ ^68^ ^]^ During evaporation, the Ag(111) crystal was either at RT or cooled to <170 K, and was quickly transferred to a precooled microscope stage (≈104 K). The samples evaporated at LT were annealed to 200 K for 10 min before measuring to avoid second layer species. The measurement parameters and preparation details can be found in Table S1, Supporting Information. The Ag(111) and Pt(111) crystals were obtained from MaTeck. Au(111) was cleaned via Ar^+^ ion sputtering and annealing to 900 K. Pt(111) was cleaned by Ar^+^ ion sputtering, followed by annealing the crystal at 1100 K for 10 min, oxidizing it under O_2_ atmosphere (1 × 10^−6^ mbar) at 800 K for 10 min, and a final annealing step at 1100 K for 10 min. The recorded STM and AFM images were processed using the Gwyddion^[^ ^77^ ^]^ software. Each image was background‐subtracted and moderately 2D‐filtered. To obtain comparable image sizes, some images were cut in size in Gwyddion and/or the CorelDraw software. The reported distances and angles were measured with the WSxM^[^ ^78^ ^]^ software. The corresponding values are shown in Table S3, Supporting Information. No drift correction was applied in the shown images as the effect has been determined as <1% for the reported measurements.

## Conflict of Interest

The authors declare no conflict of interest.

## Supporting information

Supplementary Material

## Data Availability

The data that support the findings of this study are openly available in [Zenodo] at [10.5281/zenodo.15045146], reference number [REF].
