# Supplementary material for: Flexible 2D Structure Formation of [C1C1Im][Tf2N] on Ag(111)
Source: Chemphyschem. 2025 May 25;26(13):e202500163. doi: 10.1002/cphc.202500163 (PMC12225752; doi:10.1002/cphc.202500163)
Supplement: Supplementary file 1 — Supplementary Material [file CPHC-26-e202500163-s001.pdf]

SUPPORTING INFORMATION

Flexible 2D structure formation of [C_1C_1Im][Tf_2N] on Ag(111)

Afra Gezmis ORCID: 0000-0002-9288-4872, Timo Talwar, ORCID: 0009-0002-1373-6108
Manuel Meusel ORCID: 0000-0002-3800-3894, Andreas Bayer ORCID: 0000-0002-7353-3532,
Florian Maier ORCID: 0000-0001-9725-8961, Hans Peter Steinrück* ORCID: 0000-0003-1347-8962

Lehrstuhl für Physikalische Chemie II, Universität Erlangen-Nürnberg,
Egerlandstr. 3, 91058 Erlangen, Germany

- Melting behavior of [C_1C_1Im][Tf_2N] on Ag(111) and Au(111)
- Temperature-programmed XPS of [C_1C_1Im][Tf_2N] on Ag(111) and Au(111)
- STM of [C_1C_1Im][Tf_2N] on Pt(111
- Measurement parameters and preparation details
- Unit cell parameters for the S phase of [C_1C_1Im][Tf_2N] on Ag(111)
- References

Melting behavior of $[C_1C_1Im][Tf_2N]$ on Ag(111) and Au(111)

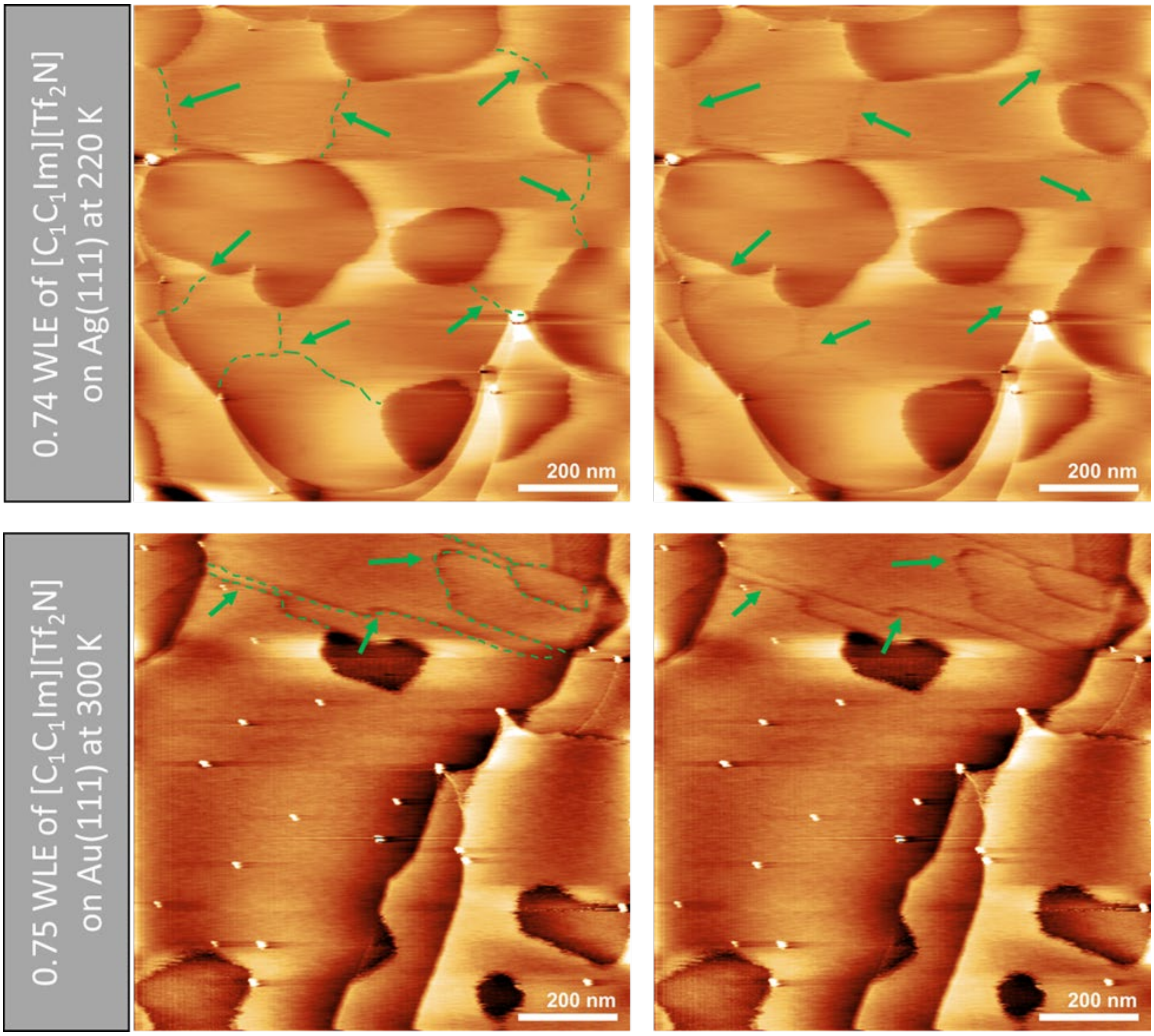

Figure S-1: AFM images (1000 nm x 1000 nm) of $[C_1C_1Im][Tf_2N]$ films of 0.74 WLE on Ag(111) (top) and 0.75 WLE on Au(111) (bottom), deposited at low temperatures (<170 K), and measured at 220 and 300 K, respectively. At these temperatures, contact lines of two or multiple domains are visible, indicating that the film is not molten yet. For both surfaces, the left and the right images are identical, but in the left dashed lines indicate the contact lines, which are difficult to see in the right. The small bright protrusions, which are mainly observed on Au(111), are attributed to defects.

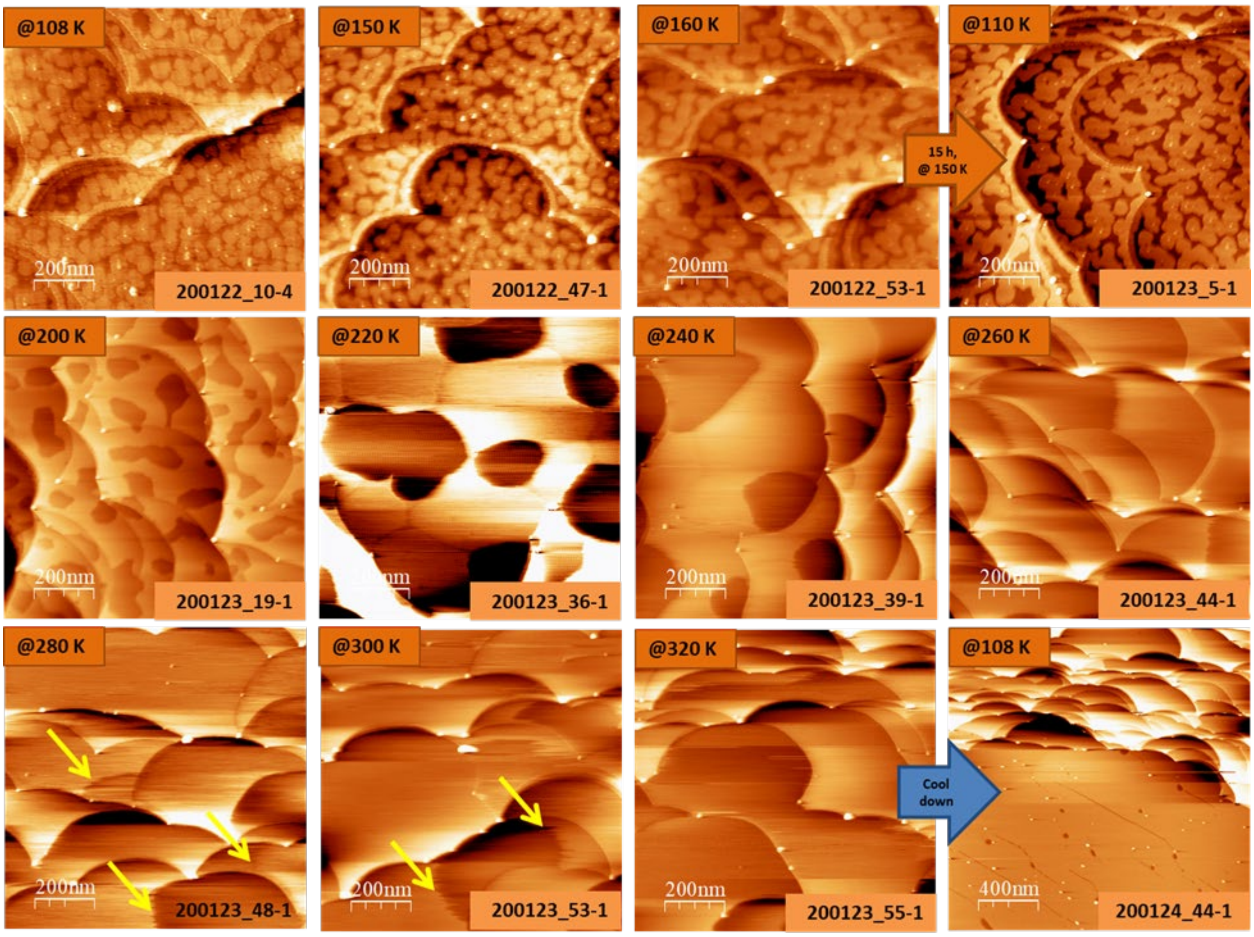

Figure S-2: Series of AFM images measured during heating of 0.74 WLE of $[C_1C_1Im][Tf_2N]$ on Ag(111), and cooling thereafter, which demonstrates the melting and freezing of the film. The film was initially deposited at <170 K; the temperature during measurement are given in top left corner of each image. Coalescence of the observed IL islands is observed starting at 200 K. Island borders, which are clearly seen with a slightly fuzzy appearance up to 260 K (see yellow arrows) are strongly washed out at 280 K and 300 K (indicated by green arrows). At 320 K, the borders of the IL film are not visible anymore. The image after cooling down to 110 K after a maximum sample temperature shows holes and cracks in the IL film indicating the IL film is still intact. From this observation it can be concluded that the WL of the IL film melts between 300-320 K, but does not desorb.

Temperature-programmed XPS of [C_1C_1Im][Tf_2N] on Ag(111) and Au(111)

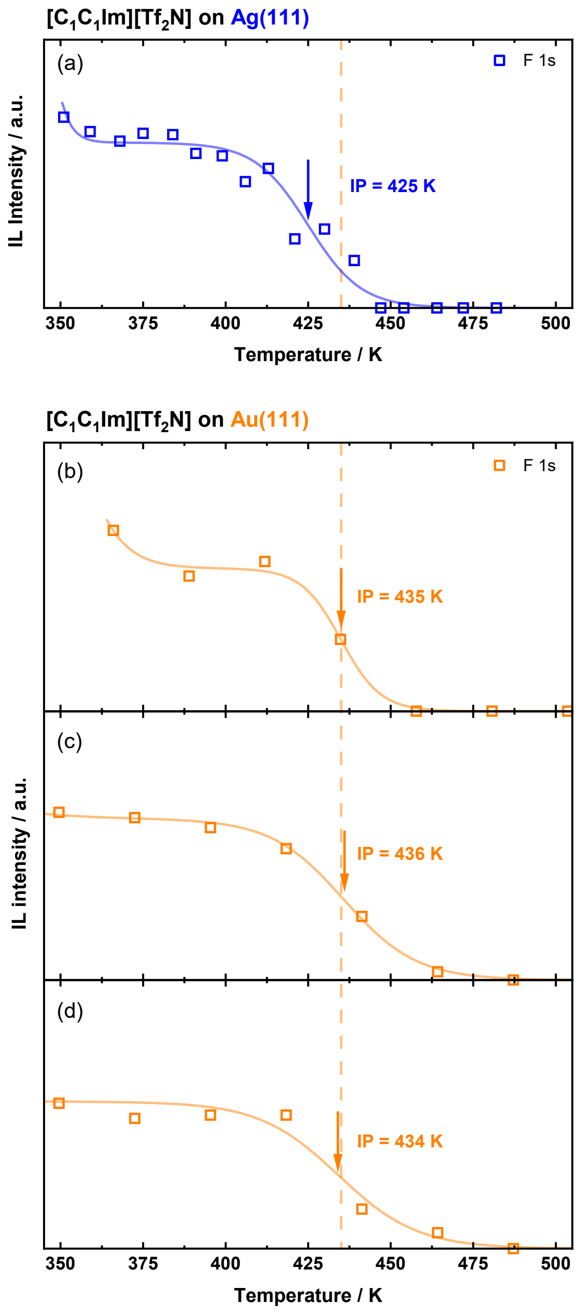

Figure S-3: Quantitative analysis of temperature-programmed X-ray photoelectron spectra (TPXPS) of the F 1s region of ~1 WL (wetting layer) of [C_1C_1Im][Tf_2N] on Ag(111) and Au(111). (a) The film on Ag(111) was prepared by heating a multilayer film deposited at room temperature to ~350 K (adapted from Ref. [3]). The films on Au(111) were prepared by depositing (b) a multilayer film, (c) ~1.0 WL, and (d) ~0.8 WL, deposited at ~100 K followed by heating to ~350 K. The heating rate during the TPXPS experiments was 2 K/s. The wetting layer desorption temperature is determined to 425 ± 2 K on Ag(111) in (a), and 435 ± 3 K on Au(111) in (b-d); the values were deduced from the inflection point of the XPS signal, where the intensity has decreased to 50% of its initial value. Notably, the C 1s spectra (not shown) on Au(111) indicate some IL decomposition, as deduced from small amounts of carbon remaining after the F 1s signals had disappeared. Since no decomposition has been previously reported for this IL/metal system, we attribute this to prolonged X-ray exposure. On Ag(111), the C 1s region was not measured, resulting in higher data-density for the F 1s region. The experimental setup used for XPS was described in Refs. 3 and 4. The IL used for the XPS measurements on Au(111) was purchased from IoLiTec, the IL used for the XPS experiments on Ag(111) was synthesized by N. Taccardi.[5]

STM of [C_1C_1Im][Tf_2N] on Pt(111)

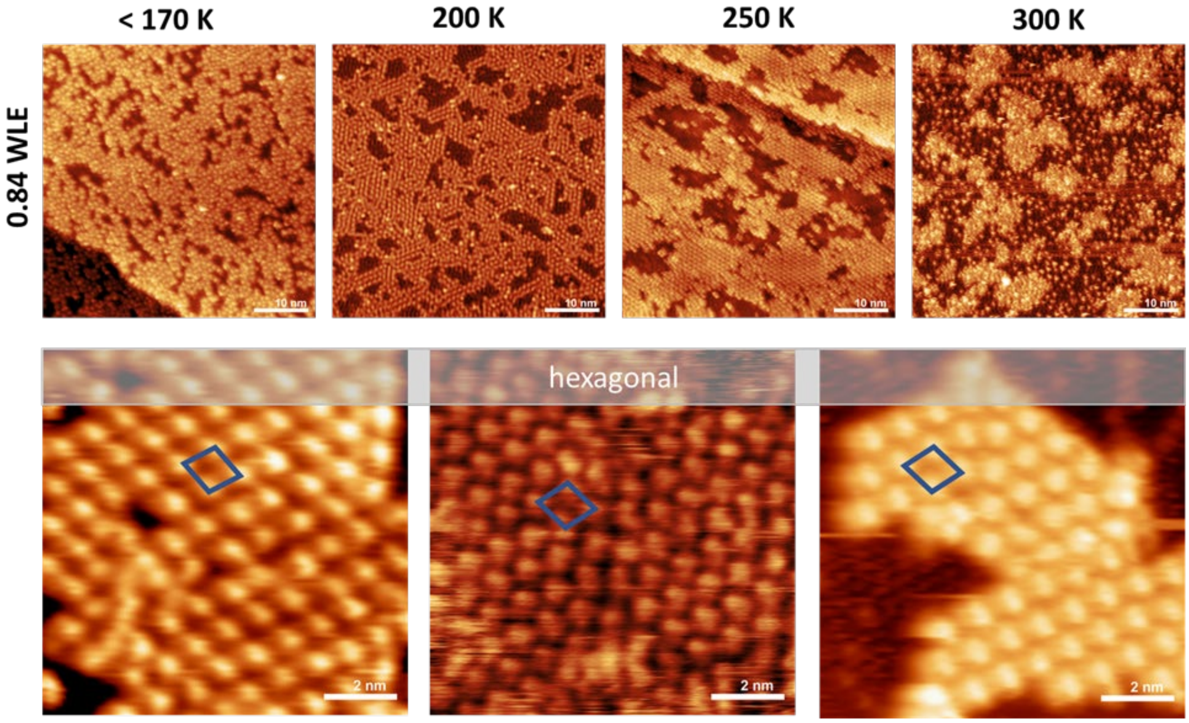

Figure S-4: *STM of [C_1C_1Im][Tf_2N] on Pt(111). (top row) 50 nm x 50 nm STM images of 0.84 WLE of* [C_1C_1Im][Tf_2N] evaporated on Pt(111) below 170 K, and cooled to <110 K afterwards. The sample was then annealed stepwise to 200, 250, and 300 K for 10 min each and cooled back down to <110 K for the measurement. The transition from a disordered WL upon deposition to ordered island can be observed upon annealing to 200 K. The islands grow further when annealed to 250 K. After annealing to 300 K only small ordered islands surrounded by disordered structures are observed. This agrees with the observations reported by Massicot *et al.* [4] (bottom row) Molecularly resolved STM images of the IL on Pt(111) for different conditions (left: 0.84 WLE, annealed to 250 K, measured <110 K; middle: 0.84 WLE, annealed to 250 K, measured 250 K; right: 0.12 WLE, annealed to 250 K, measured <110 K). All three show the same hexagonal unit cell (blue).

Measurement parameters and preparation details

Table S-1: Overview over the preparation conditions for the shown images. Typically, the preparation procedure was sputtering, annealing, deposition of IL at T_{prep} ("<" indicates that the sample was cooling during preparation), an optional annealing step to T_{max}, and finally imaging at T_{meas}. The dose is given in WLE, where 1 WLE is the dose required to complete the first layer of IL on the substrate. (*: for the IL on Cu(111) 1 WLE does not correspond to a complete full layer) The tunneling bias (U) is applied to the substrate. The frequency setpoint (Δf) is the offset to the cantilever resonance frequency.

FIGURE	*Dose [WLE]*	*Substrate*	T_S^{evap} *[K]*	T_{max} *[K]*	T_{meas} *[K]*	*U [V]*	*I [pA]*	*Δf [Hz]*	*filename*	*Note*
1 (top)	0.74	Ag(111)	129	129	108			-180	20200122_17-3	
	0.74	Ag(111)	129	200	200			-250	20200123_16-1	
	0.74	Ag(111)	129	220	220			-250	20200123_36-1	
	0.74	Ag(111)	129	300	300			-250	20200123_53-1	
1 (bottom)	0.75	Au(111)	<200	<200	110			-300	20190826_8-1	
	0.75	Au(111)	<200	200	200			-300	20190827_77-1	
	0.75	Au(111)	<200	250	250			-300	20190828_108-1	
	0.75	Au(111)	<200	300	300			-300	20190828_118-4	
2 (left)	0.77	Ag(111)	156	200	104			-400	20231019_22-1	
2 (right)	0.50	Ag(111)	156	200	104			-500	20230920_51-1	
3	0.50	Ag(111)	303	303	106	-1.0	600		20231025_17-1	
4	0.50	Ag(111)	303	303	106			-400	20230905_15-7	L
	0.50	Ag(111)	303	303	106			-400	20230905_15-3	
	0.50	Ag(111)	303	303	106			-400	20230905_15-2	
	0.50	Ag(111)	303	303	106			-400	20230905_14-1	
	0.50	Ag(111)	303	303	106			-400	20230905_11-1	
	0.50	Ag(111)	303	303	105			-600	20230906_13-3	A
	0.50	Ag(111)	303	303	105			-500	20230906_8-1	
	0.50	Ag(111)	303	303	105			-600	20230906_10-1	G
	0.50	Ag(111)	303	303	105			-600	20230906_14-3	
	0.50	Ag(111)	156	200	104			-500	20230920_35-1	
	0.50	Ag(111)	156	200	104			-500	20230920_62-5	
	0.50	Ag(111)	156	200	104			-500	20230920_54-1	
	0.50	Ag(111)	156	200	104			-500	20230920_52-1	
	0.50	Ag(111)	156	200	104			-500	20230920_35-6	
	0.50	Ag(111)	156	200	104			-500	20230920_79-1	J
	0.50	Ag(111)	156	200	104			-500	20230921_27-1	
	0.50	Ag(111)	156	200	104			-500	20230921_29-2	K
	0.50	Ag(111)	158	200	103	-1.0	600		20231025_13-4	
	0.50	Ag(111)	158	200	103	-1.0	600		20231025_15-2	
	0.50	Ag(111)	158	200	103	-1.0	600		20231025_17-1	
	0.50	Ag(111)	158	200	103	-1.0	400		20231026_7-1	
	0.50	Ag(111)	158	200	103	-0.8	300		20231026_9-6	F
	0.50	Ag(111)	158	200	103	-1.0	300		20231027_22-3	C
	0.50	Ag(111)	158	200	103	-1.0	300		20231027_22-7	D
	0.50	Ag(111)	158	200	103	-1.0	100		20231027_22-1	
	0.50	Ag(111)	158	200	103	-1.0	500		20231027_27-3	B

	0.77	Ag(111)	151	200	103	-1.0	1000		20231012_13-2	H
	0.77	Ag(111)	160	200	107	-1.0	200		20231109_15-1	
	0.77	Ag(111)	160	200	107	-1.0	200		20231109_16-1	
	0.77	Ag(111)	160	200	107	-1.0	400		20231109_28-6	
	0.77	Ag(111)	160	200	107	-1.0	300		20231109_6-1	
	0.77	Ag(111)	160	200	103	-0.7	400		20231110_9-2	
	0.77	Ag(111)	160	200	103	-0.7	400		20231110_11-2	
	0.94	Ag(111)	165	200	104	1.0	300		20231122_5-1	E
	0.94	Ag(111)	165	200	104	1.0	300		20231122_11-1	
	0.94	Ag(111)	165	200	104	1.0	300		20231122_11-5	
	0.94	Ag(111)	165	200	104	1.0	300		20231122_13-1	
	0.94	Ag(111)	160	200	104	1.0	300		20231212_3-1	
	0.94	Ag(111)	160	200	104	1.0	200		20231212_5-1	
	0.94	Ag(111)	160	200	104	1.0	200		20231212_4-2	
	0.94	Ag(111)	160	200	104	1.0	200		20231212_11-3	
	0.94	Ag(111)	160	200	103	0.8	400		20231213_16-1	
	0.94	Ag(111)	160	200	103	1.0	300		20231213_40-1	I
5	0.94	Ag(111)	160	300	103	-0.8	80		20231214_17-2	
6 (H)	1.00	Au(111)	381	381	110	-1.4	35		20181119_153-5	Adapted from[1]
6 (S)	0.48	Au(111)	168	168	110	-1.5	100		20190712_11-2	
6 (H)	0.94	Ag(111)	160	300	103	-0.8	80		20231214_17-2	
6 (S)	0.50	Ag(111)	303	303	106	-1.0	600		20231025_17-1	
6 (H)	0.30*	Cu(111)	295	295	110			-400	20201130_73-1	Adapted from[2]
6 (S)	0.30*	Cu(111)	295	295	110			-400	20201126_27-3	Adapted from[2]
6 (H)	0.84	Pt(111)	250	250	250	1.2	500		20210906_33-2	
S-1 (top)	0.74	Ag(111)	129	220	220			-250	20200123_36-1	
S-1 (bottom)	0.75	Au(111)	<200	300	300			-300	20190828_118-4	
S-2	0.73	Ag(111)	129	129	108			-200	20200122_10-4	
	0.73	Ag(111)	129	150	150			-175	20200122_47-4	
	0.73	Ag(111)	129	160	160			-175	20200122_53-1	
	0.73	Ag(111)	129	160	110			-250	20200123_5-1	
	0.73	Ag(111)	129	200	200			-250	20200123_19-1	
	0.73	Ag(111)	129	220	220			-250	20200123_36-1	
	0.73	Ag(111)	129	240	240			-250	20200123_39-1	
	0.73	Ag(111)	129	260	260			-250	20200123_44-1	
	0.73	Ag(111)	129	280	280			-250	20200123_48-1	
	0.73	Ag(111)	129	300	300			-250	20200123_53-1	
	0.73	Ag(111)	129	320	320			-250	20200123_55-1	
	0.73	Ag(111)	129	320	108			-160	20200124_44-1	
S-4 (top)	0.84	Pt(111)	159	159	109	1.2	100		20210713_31-1	
	0.84	Pt(111)	159	200	109	1.2	100		20210714_18-6	
	0.84	Pt(111)	159	250	109	1.2	1000		20210715_40-3	
	0.84	Pt(111)	159	300	109	1.2	1000		20210716_55-4	
S-4 (bottom,l)	0.84	Pt(111)	250	250	111	1.2	300		20210909_95-1	
S-4 (bottom,m)	0.84	Pt(111)	250	250	250	1.2	500		20210906_33-2	
S-4 (bottom,r)	0.12	Pt(111)	158	250	110	1.2	670		20210804_113-1	

Table S-2: Explanation of measurement sets shown in Figure 4, which vary in vary in evaporation temperature (RT: empty symbols, LT (<170 K): full symbols), coverage or measurement type (STM: square, or AFM: triangle).

SYMBOL	SET NUMBER	EVAPORATION TEMPERATURE	COVERAGE	MEASUREMENT TECHNIQUE	H PHASE FOUND
△	1	303 K	0.50 WLE	AFM	NO
▲	2	155 K	0.50 WLE	AFM	NO
■	3	158 K	0.50 WLE	STM	YES
▲	4	151-156 K	0.77 WLE	AFM	YES
■	5	160 K	0.77 WLE	STM	NO
■	6	159-160 K	0.94 WLE	STM	YES

Distance and area parameters for the S phase of $[C_1C_1Im][Tf_2N]$ on Ag(111)

Table S-3: Distances $|\vec{a}|$ and $|\vec{s}|$ and the enclosed areas for the analysis of the striped phase of the IL formed on Ag(111). For preparation and measurement parameters, see Table S-1.

FILENAME	$\vert\vec{a}\vert$ [nm]	$\vert\vec{s}\vert$ [nm]	*area* [nm^2]
20230905_15-7	1.21	0.82	0.99
20230905_15-3	1.18	0.94	1.11
20230905_15-2	1.14	0.92	1.05
20230905_14-1	1.14	0.96	1.09
20230905_11-1	1.09	0.98	1.07
20230906_13-3	0.92	0.70	0.64
20230906_8-1	1.32	0.86	1.14
20230906_10-1	0.92	0.81	0.75
20230906_14-3	1.13	0.70	0.79
20230920_35-1	1.00	0.97	0.97
20230920_62-5	0.74	1.32	0.98
20230920_54-1	0.81	1.18	0.96
20230920_52-1	0.65	1.19	0.77
20230920_35-6	0.88	0.97	0.85
20230920_79-1	1.06	0.83	0.88
20230921_27-1	1.06	0.77	0.82
20230921_29-2	1.16	0.69	0.80
20231025_13-4	0.81	0.94	0.76
20231025_15-2	0.81	0.99	0.80
20231025_17-1	0.87	0.91	0.79
20231026_7-1	0.77	1.13	0.87
20231026_9-6	0.75	1.21	0.91
20231027_22-3	0.85	0.95	0.81
20231027_22-7	0.82	1.03	0.84
20231027_22-1	0.80	1.05	0.84
20231027_27-3	0.77	0.81	0.62
20231012_13-2	0.97	0.92	0.89
20231109_15-1	0.98	0.90	0.88
20231109_16-1	0.93	0.81	0.75
20231109_28-6	0.85	0.94	0.80
20231109_6-1	0.77	0.85	0.65
20231110_9-2	0.56	0.90	0.50
20231110_11-2	0.70	0.74	0.52
20231122_5-1	0.68	1.05	0.71
20231122_11-1	0.82	0.91	0.75
20231122_11-5	0.85	0.92	0.78
20231122_13-1	0.67	0.93	0.62
20231212_3-1	0.64	0.90	0.58
20231212_5-1	0.87	0.65	0.57
20231212_4-2	0.86	0.70	0.60
20231212_11-3	0.65	0.93	0.60
20231213_16-1	0.90	0.90	0.81
20231213_40-1	0.99	0.93	0.92

References

(1) Meusel, M.; Lexow, M.; Gezmis, A.; Schötz, S.; Wagner, M.; Bayer, A.; Maier, F.; Steinrück, H.-P. Atomic Force and Scanning Tunneling Microscopy of Ordered Ionic Liquid Wetting Layers from 110 K up to Room Temperature. *ACS Nano* **2020**, *14* (7), 9000-9010. DOI: 10.1021/acsnano.0c03841.

(2) Adhikari, R.; Massicot, S.; Fromm, L.; Talwar, T.; Gezmis, A.; Meusel, M.; Bayer, A.; Jaekel, S.; Maier, F.; Görling, A.; et al. Structure and Reactivity of the Ionic Liquid [C1C1Im][Tf2N] on Cu(111). *Topics in Catalysis* **2023**, *66* (15), 1178-1195. DOI: 10.1007/s11244-023-01801-y.

(3) Lexow, M.; Maier, F.; Steinrück, H.-P. Ultrathin ionic liquid films on metal surfaces: adsorption, growth, stability and exchange phenomena. *Advances in Physics: X* **2020**, *5* (1), 1761266. DOI: 10.1080/23746149.2020.1761266.

(4) Massicot, S.; Gezmis, A.; Talwar, T.; Meusel, M.; Jaekel, S.; Adhikari, R.; Winter, L.; Fernández, C. C.; Bayer, A.; Maier, F.; et al. Adsorption and thermal evolution of [C1C1Im][Tf2N] on Pt(111). *Physical Chemistry Chemical Physics* **2023**, *25* (41), 27953-27966, 10.1039/D3CP02743K. DOI: 10.1039/D3CP02743K.

(5) Lovelock, K. R. J.; Kolbeck, C.; Cremer, T.; Paape, N.; Schulz, P. S.; Wasserscheid, P.; Maier, F.; Steinrück, H. P. Influence of Different Substituents on the Surface Composition of Ionic Liquids Studied Using ARXPS. The Journal of Physical Chemistry B 2009, 113 (9), 2854-2864. DOI: 10.1021/jp810637d.
